# Shoulder Pain, Functional Status, and Health-Related Quality of Life after Head and Neck Cancer Surgery

**DOI:** 10.1155/2013/601768

**Published:** 2013-12-25

**Authors:** Hsiao-Lan Wang, Juanita F. Keck, Michael T. Weaver, Alan Mikesky, Karen Bunnell, Janice M. Buelow, Susan M. Rawl

**Affiliations:** ^1^University of South Florida College of Nursing, 12901 Bruce B. Downs Boulevard, MDC 22, Tampa, FL 33612, USA; ^2^Indiana University School of Nursing, Indianapolis, IN 46202, USA; ^3^Indiana University School of Physical Education & Tourism Management, Indianapolis, IN 46202, USA; ^4^Head and Neck Surgery Program, Saint Vincent Indianapolis Hospital, Indianapolis, IN 46260, USA; ^5^Center for Ears, Nose, Throat, and Allergy, Carmel, IN 46032, USA

## Abstract

Head and neck cancer (HNC) patients experience treatment-related complications that may interfere with health-related quality of life (HRQOL). The purpose of this study was to describe the symptom experience (shoulder pain) and functional status factors that are related to global and domain-specific HRQOL at one month after HNC surgery. In this exploratory study, we examined 29 patients. The outcome variables included global HRQOL as well as physical, functional, emotional, and social well-being. Symptom experience and functional status factors were the independent variables. In the symptom experience variables, shoulder pain distress was negatively associated with physical well-being (*R* ^2^ = 0.24). Among the functional status variables, eating impairment was negatively related to global HRQOL (*R* ^2^ = 0.18) and physical well-being (*R* ^2^ = 0.21). Speaking impairment and impaired body image explained a large amount of the variance in functional well-being (*R* ^2^ = 0.45). This study provided initial results regarding symptom experience and functional status factors related to poor HRQOL in the early postoperative period for HNC patients.

## 1. Introduction

Up to 80% of head and neck cancer (HNC) patients who had neck lymph node dissection experienced shoulder pain that led to impaired shoulder function [1–3]. Cancers of the head and neck include malignant tumors of the buccal cavity, larynx, pharynx, thyroid, salivary glands, and nose/nasal passages [4]. Because of the specific anatomic structures involved, HNC treatment negatively impacts one or more body functions such as breathing [5, 6], eating [7, 8], speaking [8, 9], and body image [9, 10]. There are 113,860 new cases of head and neck cancer (HNC) expected in 2013 [11].

Shoulder pain, impaired shoulder function, impaired body image, and difficulty with breathing, eating, or speaking contribute to decrements in health-related quality of life (HRQOL) [12, 13]. In addition to survival and recurrence, HRQOL has been considered one of the most important outcomes in HNC studies [14]. Although there is no standard instrument to measure HRQOL, the majority of researchers agree that HRQOL is a subjective and multidimensional construct consisting of four main domains in a person's health-related life: physical well-being, functional well-being, emotional well-being, and social well-being [15–17].

Longitudinal studies have shown that HRQOL in patients with HNC declined immediately after cancer treatment when compared with pretreatment baseline data [12, 18–20]. Early rehabilitation has been suggested as an important strategy to help HNC patients cope with and adjust to the long-term effects of cancer treatments [21]. However, before interventions can be designed to enhance HRQOL in the early postoperative period, descriptive research is needed to inform clinicians about the importance of identifying HNC patients with shoulder pain and impaired body functions, both of which put them at risk for poor HRQOL.

**Figure 1:**
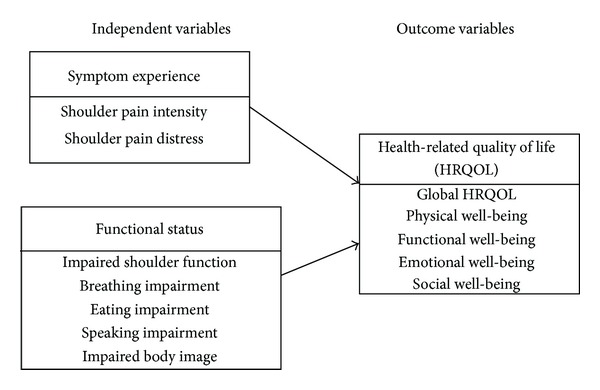
Conceptual Framework.

The conceptual framework of this study was built from the University of California, San Francisco School of Nursing Symptom Management Model (UCSF-SMM) and empirical evidence (Figure 1). Researchers have developed the UCSF-SMM to describe “symptom experience” and “outcomes” as two interrelated dimensions [22, 23]. “Symptom experience” has been conceptualized as symptom intensity and symptom distress in previous research [24, 25], while “symptom experience” has also been studied as a single symptom [25–27] or as multiple symptoms [24, 28–30]. “Outcomes” that have been found to be related to “symptom experience” in studies include functional status [28] and quality of life [24, 26, 27], which can be influenced by each other [22]. In the HNC research, functional status has been operationalized by the International Classification of Functioning, Disability and Health (ICF) of the World Health Organization [31–35]. The Centers for Disease Control and Prevention (CDC) stated that self-reported multidimensional HRQOL is a valid indicator of disease or treatment outcomes [36].

It is well known that the temporary or permanent denervation of the trapezius muscle secondary to spinal accessory nerve injury during neck dissection results in shoulder pain and impaired shoulder function [37]. We used neck dissection as one of the inclusion criteria. Our study applied concepts of the UCSF-SMM and empirical evidence to describe the relationships among shoulder pain, impaired body functions, and HRQOL after HNC surgery. The focused outcome is HRQOL in our study. Therefore, the outcome variables consisted of global HRQOL and four domain-specific HRQOL, including physical well-being, functional well-being, emotional well-being, and social well-being. The independent variables were categorized as symptom experience and functional status factors. Symptom experience was defined as shoulder pain experience after HNC surgery. Symptom experience factors included shoulder pain intensity and shoulder pain distress in our study. Functional status was defined as impaired body functions after HNC surgery. In ICF, body functions are the physiological and psychological functions of body systems, and impairments are deviations or losses in body functions [38]. Both patient self-reporting and provider-reported impairments of body functions were documented in previous HNC studies using ICF [34, 35, 39]. In our study, functional status, which is impaired body functions, consisted of impaired shoulder function (ICF Code: b710 impaired mobility of joint functions), breathing impairment (ICF Code: b440 imparted respiration functions), eating impairment (ICF Code: b510 impaired ingestion functions), speaking impairment (ICF Codes: b310 impaired voice functions and b320 impaired articulation functions), and impaired body image (ICF Code: b180 impaired experience of self and time functions) [31]. Limited shoulder abduction was identified as the most frequently seen impaired shoulder function after neck dissection surgery [37].

The purpose of this study was to describe the shoulder symptom experience and functional status factors related to HRQOL at one month after HNC surgery (early postoperative period). Our study was designed to answer the following research questions.

1. What are the bivariate associations between selected shoulder symptom experience and functional status factors and (a) global HRQOL and (b) domain-specific HRQOL (physical, functional, emotional, and social well-being) at one month after HNC surgery? Shoulder symptom experience factors were shoulder pain intensity and shoulder pain distress. Functional status factors consisted of impaired body functions (i.e., limited shoulder abduction, impaired body image, and eating, speaking, and breathing impairments).
2. What are the relationships between the independent variables and outcome variables in the proposed conceptual framework? The outcome variables included global and domain-specific HRQOL. Symptom experience and functional status factors were examined as independent variables.

## 2. Materials and Methods

### 2.1. Sample and Setting

This descriptive, correlational study was conducted with a convenience sample of 29 head and neck cancer patients recruited from a midwestern hospital. The study was approved by the University and Hospital Institutional Review Boards. Data were collected via self-administered surveys and physical assessments at one month after HNC surgery. Inclusion criteria were that participants (a) had received their first neck dissection surgery, (b) were able to understand English, and (c) were able to provide informed consent. Patients who had preexisting shoulder pain or limited shoulder range of motion prior to surgery were excluded.

### 2.2. Measures

Data on age, gender, race, marital status, educational background, and job status were collected using a demographic survey. Participants were asked to self-report on their current use of tobacco and alcohol. Primary cancer site and cancer stage were extracted from medical records. A self-administered survey and physical exam were applied to collect the outcome and independent variables.

#### 2.2.1. Outcome Variables

HRQOL was measured using the 26-item general scale of Functional Assessment of Chronic Illness Therapy-Head and Neck Scale, version 4 (FACIT-H&N) [40, 41]. The items comprised four domain-specific subscales: physical well-being (6 items, no pain item), emotional well-being (6 items), functional well-being (7 items), and social well-being (7 items). Participants were asked to indicate how true each statement (item) was for them during the previous 7 days by responding to a 5-point Likert scale (0 = * not at all*, 1 = * a little bit*, 2 = * some what*, 3 = * quite a bit*, and 4 = * very much*). Higher scores meant better HRQOL. Global HRQOL was computed by summing the score for each of the four subscales. Internal consistency reliability was evaluated using Cronbach's alpha and was found to be 0.84 for the 26-item general scale, 0.68 for the physical subscales, 0.68 for the emotional subscale, 0.79 for the functional subscale, and 0.69 for the social subscale.

#### 2.2.2. Independent Variables

Shoulder pain intensity was measured using four severity items from the Brief Pain Inventory (BPI) [42, 43]. These items assessed shoulder pain at its worst, at its least, on average in the past week, and at the time of the interview, using an 11-point scale where 0 = * no pain* and 10 = * pain as bad as you can imagine*. Cronbach's alpha coefficient for the four BPI severity items was 0.90 in this sample. Shoulder pain distress was measured using an 11-point numeric rating scale where 0 = * no distress* and 10 = * most distress imaginable* [44]. Participants marked the number representing the amount of distress they had experienced from shoulder pain in the previous week.

Shoulder function was defined as the maximum degree of shoulder abduction possible as measured by a 12-inch 360-degree goniometer. Limited shoulder abduction, that is, less than 180°, was considered as impaired shoulder function [45]. The principal investigator was trained by a physical therapist to measure the shoulder abduction. After training, interrater reliability between the investigator and the physical therapist was established using 10 healthy adults (*r* = 0.87). If participants had a unilateral neck dissection, shoulder abduction was measured on the surgical side, while for those who had bilateral neck dissections, the mean degree of shoulder abduction on both sides was calculated.

Other impaired body functions were operationalized by the head and neck subscale of FACIT-H&N. This subscale included 9 items to measure impaired body image (1 item), as well as impairments in breathing (1 item), eating (5 items), and speaking (2 items). The selected items were conceptually and statistically related to each respective construct [46]. Impaired body image was measured using a single item: “I am unhappy with how my face and neck look.” Another single item measured breathing impairment: “I have trouble breathing normally.” Five items measured eating impairment, including “I am able to eat the foods that I like,” “My mouth is dry,” “I am able to eat as much food as I want,” “I can swallow naturally and easily,” and “I can eat solid foods ” (*α* = 0.80). The two items that measured speaking impairment were “My voice has its usual quality and strength” and “I am able to communicate with others” (*α* = 0.72). Participants were asked to rate how true each item had been for them during the previous 7 days by responding to the same 5-point Likert scale in the general scale. Scales were transformed so that a higher score indicated greater impairment.

### 2.3. Procedure

This study was approved by both the University and Healthcare Service Institutional Review Boards. A nurse practitioner who was responsible for direct care of this patient population identified potentially eligible participants who had undergone HNC surgery and introduced them to the study. Patients who gave permission to be approached were visited in their private hospital room by the investigator within 24 to 48 hours prior to being discharged. The investigator explained the study, answered questions, and assessed the interest in participating. Of the 34 patients who expressed interest, 29 (85%) were eligible and they enrolled. An appointment was then set for data to be collected in conjunction with a regular 30-day follow-up visit with their surgeon. At this follow-up clinic visit, participants completed the self-administered surveys, and their shoulder abduction was evaluated. Participants received a $25 gift card as compensation for their time. The medical record review was completed at the hospital or in the surgeon's office.

### 2.4. Statistical Analyses

Data were analyzed using PASW 18.0 (SPSS Inc, Chicago, IL). Descriptive statistics were computed to describe the demographic and clinical variables and assess the distribution of independent and outcome variables. For the first research question, the bivariate associations among the symptom experience and functional status variables and five HRQOL variables were examined using Pearson correlation coefficients. For the second research question, separate multiple regression models were examined for each of the five HRQOL outcome variables. Stepwise elimination was employed to identify independent variables in each category (symptom experience or functional status) that were significantly related to HRQOL variables, based on a 5% Type I error rate.

## 3. Results

The descriptive statistics for the demographic and clinical variables are shown in Table 1. Two thirds of the participants were male, 93% were Caucasian, 42% had cancer of the lip or oral cavity, and 59% had stage IV cancer. The majority were not currently using tobacco (86%) or alcohol (86%). Their mean age was 60.34 years and they ranged in age from 36 to 89 years old.

Table 2 displays the descriptive statistics and possible ranges for the outcome and independent variables. The highest mean score among the outcome (HRQOL) variables was on the social well-being subscale (*M* = 3.40 ± 0.70), while the lowest was on the functional well-being subscale (*M* = 2.55 ± 0.86). On average, participants in our study reported mild shoulder pain intensity and shoulder pain distress. Thirty-eight percent (*n* = 11) did not report any shoulder pain. Mean shoulder abduction was 111.59 degrees (SD = 27.27). Participants reported having greater eating impairment (*M* = 2.08 ± 1.11) than breathing impairment (*M* = 0.76 ± 1.18), speaking impairment (*M* = 1.76 ± 1.01), or impaired body image (*M* = 1.38 ± 1.59).

### 3.1. Research Question 1: What Are the Bivariate Associations between Selected Symptom Experience and Functional Status Factors and (a) Global HRQOL and (b) Domain-Specific HRQOL (Physical, Functional, Emotional, and Social Well-Being) at One Month after HNC Surgery?

We initially examined the relationships among the independent variables under the symptom experience and functional status categories, respectively. Regarding symptom experience variables, shoulder pain intensity and shoulder pain distress were positively correlated with each other (*r* = 0.92, *P* < 0.01). For the functional status variables, lower degrees of shoulder abduction were associated with higher impaired body image scores (*r* = −0.39, *P* = 0.04). Higher eating impairment scores were related to lower degrees of shoulder abduction (*r* = −0.45, *P* = 0.01) and higher speaking impairment scores (*r* = 0.45, *P* = 0.02).

To answer Research Question 1, we conducted bivariate associations among symptom experience, functional status, and HRQOL variables (Table 3). Both symptom experience variables were inversely related to physical well-being, indicating that participants with more severe shoulder pain intensity (*r* = −0.42, *P* = 0.02) or shoulder pain distress (*r* = −0.49, *P* = 0.01) had lower physical well-being scores. Moreover, four out of five functional status variables were significantly associated with functional well-being. Lower functional well-being scores were related to lower degrees of shoulder abduction (*r* = 0.46, *P* = 0.01) and higher eating impairment (*r* = −0.53, *P* < 0.01), speaking impairment (*r* = −0.56, *P* < 0.01), and impaired body image (*r* = −0.45, *P* < 0.01) scores. In addition to functional well-being, global HRQOL (*r* = −0.42, *P* = 0.02) and physical well-being (*r* = −0.45, *P* = 0.01) scores were also negatively correlated with eating impairment. In other words, participants who experienced severe eating impairment reported lower global HRQOL and physical well-being scores.

### 3.2. Research Question 2: What Are the Relationships between the Independent Variables and Outcome Variables in the Proposed Conceptual Framework? The Outcome Variables Included Global and Domain-Specific HRQOL. Symptom Experience and Functional Status Factors Were Examined as Independent Variables

Table 4 presents the results from the multiple regression models that were used to examine the relationships between the independent variables and outcome variables depicted in the conceptual framework. When symptom experience variables were entered as independent variables to predict both global and domain-specific HRQOL variables, shoulder pain distress was identified as a significant predictor of physical well-being, with 24% of the variance explained (*F*(1,27) = 8.44, *P* = 0.01). Shoulder pain intensity, the other symptom experience factor, was not significant in this model. Based on our conceptual framework, no other outcome variables were significantly associated with symptom experience factors.

Next, functional status variables were entered as independent variables to predict each HRQOL outcome variable in the regression analyses. Eating impairment was consistently predictive of global HRQOL and physical well-being. Specifically, eating impairment accounted for 18% of the variance in global HRQOL (*F*(1,27) = 5.80, *P* = 0.02) and 21% of the variance in physical well-being (*F*(1,27) = 7.02, *P* = 0.01). In addition, two functional status variables, speaking impairment and impaired body image, explained 45% of the variance in functional well-being (*F*(2,26) = 10.59, *P* < 0.01). None of the symptom experience or functional status variables were significant predictors of emotional or social well-being.

## 4. Discussion

This study provided preliminary data regarding the symptom experience and functional status factors that are related to HRQOL in the early postoperative period among HNC patients. The results also partially supported the conceptual framework we proposed based on the UCSF-SMM and empirical evidence. The findings revealed that patients with more severe shoulder pain distress, impaired eating and speaking functions, and impaired body image could be at risk for reduced levels of HRQOL, especially in physical and functional well-being.

**Figure 2:**
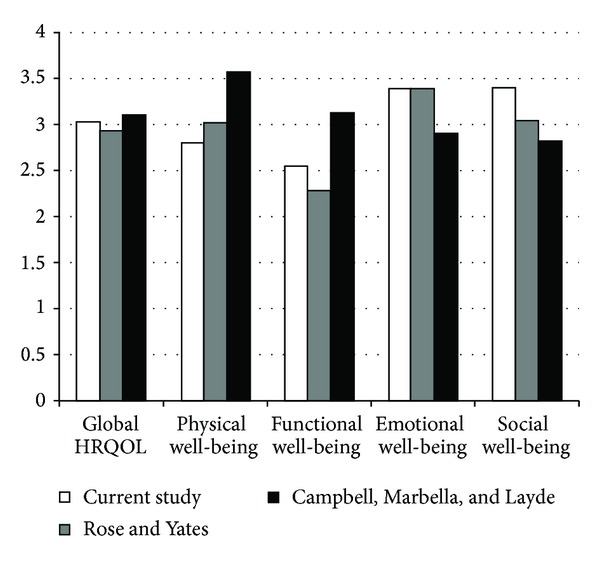
Means of HRQOL variables from the FACIT among three studies. Participants in these studies had various types of HNC. Data collection in our study was one month after HNC surgery; data collection in the study by Rose and Yates [47] was one month after radiation; and data collection in the study by Campbell et al. [48] was more than 3 years after surgery and/or radiation.

Figure 2 shows the means of the HRQOL variables of the current study and two other studies [47, 48]. Participants in these studies had various types of HNC. Our study was conducted at one month after HNC surgery, the study by Rose and Yates [47] was conducted at one month after radiation, and the study by Campbell et al. [48] was conducted at over 3 years after surgery and/or radiation. The global HRQOL scores were similar across these three studies. Our study and the study by Rose and Yates showed that the participants in the early period of post-HNC treatments had worse physical and functional well-being scores than emotional and social well-being scores. However, the long-term survivor participants (>3 years) in the study by Campbell et al. reported worse emotional and social well-being scores than physical and functional well-being scores. These findings indicate that HNC patients had perhaps expected that they would encounter multiple problems related to their surgery and/or radiation in the early posttreatment period. Patient education and communication with healthcare providers before the treatment possibly prepared patients to cope with the symptom and impaired body functions. Social support from family and friends potentially remained strong because of their hope of receiving effective results from the treatment. Therefore, these could explain why symptom experience and functional status factors did not predict emotional or social well-being. On the other hand, long-term HNC survivors experienced recovery of physical and functional well-being but reported worse emotional and social well-being compared with the survivors in the early posttreatment period. Since predictors of global and domain-specific HRQOL may change over time, more longitudinal studies are needed.

The most robust finding in our study was that 45% of the variance in functional well-being was explained by speaking impairment and impaired body image. Participants with greater speaking impairment and more impaired body image reported worse functional well-being at one month after HNC surgery. One study with long-term HNC survivor patients (>5 years) found no relationship between speaking impairment and functional well-being [49]. It is possible that our participants were still adjusting to their impaired speech in the early postoperative period. Loss of voice or intelligible speech interferes with communication [9]. Psychological distress and feelings of social isolation resulting from lack of communication have been well documented [9, 50]. Augmentive and alternative communication devices, such as pencil-paper instruments or preprogrammed message boards, have been suggested as communication aids for patients after HNC surgery [9, 21]. In our study, speaking impairment was not significantly associated with emotional well-being. This finding implies that participants perhaps adapted to the speaking impairment by use of augmentive and alternative devices in the early postoperative period or that they were optimistic about improvement over time.

Previous studies showed that impaired body image was related to greater anxiety, worse relationship with a partner, impaired sexual function, and increased social isolation among HNC survivors [13, 51]. While we did not find significant relationships between impaired body image and emotional or social well-being, we did find that participants with impaired body image reported lower functional well-being. One study showed that self-care was a significant factor that reduced anxiety among HNC patients with impaired body image [52]. Therefore, it is necessary to understand whether impaired body image interferes with functional well-being that is related to conducting self-care activities in the early postoperative time.

Another important finding in our study was that eating impairment was a predictor of both global HRQOL and physical well-being. Participants with eating impairment reported lower global HRQOL and physical well-being. Similar results were seen in studies using the FACIT to measure HRQOL among HNC patients at 12 months after chemoradiation [53] and among patients with laryngeal cancer [54]. Previous studies also found that HNC patients with swallowing and chewing problems reported lower HRQOL [55, 56]. The presence of a feeding tube has been shown to be the most important predictor of reduced HRQOL [57]. The mean score for eating impairment in our study was the highest, indicating significant impairment, compared with breathing and speaking impairment and impaired body image, using the same 0–4 scale. This finding highlights the potential for eating impairment to be the greatest challenge that patients face at one month after HNC surgery.

Among symptom experience factors, shoulder pain distress was a predictor of physical well-being. Participants who reported higher distress from shoulder pain also reported lower physical well-being at one month after HNC surgery. While shoulder pain intensity was negatively correlated with physical well-being, this relationship was not significant. Among functional status factors, the association between limited shoulder abduction with impaired body image and eating impairment indicated extensive soft tissue involvement during the HNC surgery. Limited shoulder abduction was associated with poor functional well-being, although limited shoulder abduction was not a significant predictor of HRQOL when entered with other functional status factors into the multivariate model. One prior study showed that shoulder range of motion was positively associated with physical well-being among 5-year HNC survivor patients [58]. These findings suggest that shoulder pain could cause participants to avoid using their shoulders in the early postoperative period, and so limited shoulder abduction could lead to long-term impairment. The pathway from shoulder pain to impaired shoulder function, which reduces physical well-being over time, addresses the importance of early shoulder rehabilitation while shoulder pain is the primary manifestation. There is a need to examine these relationships and how they may change over time using longitudinal designs. In addition to shoulder pain, evaluation of complete shoulder range of motion (forward elevation, extension, abduction, adduction, and internal and external rotation), strength, and coordination are needed in future studies to fully understand the impaired mobility of shoulder joints among patients after HNC surgery.

The strength of our study is that the identified factors related to HRQOL reflect multidisciplinary components for post-HNC surgery care [59]. Proper pain management of shoulder pain to help HNC patients engage in physical therapy so as to prevent future impaired shoulder function due to “non-use” should be part of routine care [1, 60]. For eating impairment, patients should be weighed on a regular basis and have their nutrition intake monitored by the mouth or feeding tube. Nutritional counseling with a dietician is needed for problems with chewing and swallowing or for implementation of enteral nutrition [21, 61]. A speech-language pathologist and occupational therapist are responsible to promote early rehabilitation of speaking and swallowing functions [21]. For HNC patients with laryngectomy, tracheoesophageal puncture for a valve prosthesis placement has been considered standard care for voice rehabilitation [62]. The speech-language pathologist plays an important role in instructing and coaching the patient to use the prosthetic valve [9, 21]. Finally, our study has shown the importance of impaired body image in functional well-being in the early recovery period after HNC surgery. Patients should be encouraged to participate fully in rehabilitation programs and perform self-care activities to adjust to their new appearance [10].

Although our findings provide valuable perspectives on clinical implications for caring for HNC patients after cancer surgery, some limitations should be noted. This study is limited by the small sample size recruited from one clinical practice. Therefore, the results may not be generalizable to other HNC patients after surgery. The predictors identified in our study were only ascertained in their relationships to HRQOL outcome variables using multiple regression models. The cross-sectional design cannot explain the underlying causal mechanisms. Therefore, a prospective longitudinal study with a larger sample size is warranted to (a) examine patterns of change in symptom experience (shoulder pain) and functional status over time, (b) elicit factors that influence symptom experience and functional status, and (c) describe the types of multidisciplinary care that could be tested to improve HRQOL after HNC surgery. Finally, we proposed a limited conceptual framework which only included shoulder pain and HNC related impaired body functions as independent variables. There are likely to be other variables that may impact HRQOL but they were not examined in our study, such as pretreatment or current physical activity level, perceived stiffness, muscle weakness, and fatigue.

## 5. Conclusions

In summary, our study described HNC patients who encountered shoulder pain, eating impairment, speaking impairment, and impaired body image which interfered with HQOL during the early postoperative period. The American Cancer Society in 2013 recommended early and regular assessment of symptoms and impaired body functions across the cancer care continuum [63]. Rehabilitation diagnosis and intervention should be done by certain healthcare professionals. Among HNC patients, effective management of shoulder pain and impaired eating/speaking functions as well as adjustment of impaired body image are vital to improve HRQOL. They require a multidisciplinary care team, including a physical therapist, occupational therapist, speech-language pathologist, and dietician. The oncologist, primary care physician, and nurse should use proper tools to identify patients' symptoms and impaired body functions so as to be able to refer them to rehabilitation specialists.

## Figures and Tables

**Table 1:** Descriptive statistics of demographic and clinical variables.

Variables	Mean (SD)	Frequency (%)
Age	60.34 (12.43)	
Gender		
Male		21 (72)
Female		8 (28)
Race		
Caucasian		27 (93)
African American		2 (7)
Marital status		
Married		16 (56)
Not married		13 (44)
Educational background		
More than HS		10 (34)
HS/GED		11 (38)
Less than HS		8 (28)
Job Status		
Employed		11 (38)
Retired		11 (38)
Unemployed		7 (24)
Primary cancer site		
Lip and oral cavity		12 (42)
Pharynx		3 (10)
Larynx		9 (31)
Salivary glands		2 (7)
Thyroid		2 (7)
Ear		1 (3)
TNM cancer stage		
Stage 0		1 (3)
Stage I		2 (7)
Stage II		4 (14)
Stage III		5 (17)
Stage IV		17 (59)
Current use of tobacco		4 (14)
Current use of alcohol		4 (14)

GED: general educational development; HS: high school; and TNM: tumor, regional lymph nodes, metastasis.

**Table 2:** Descriptive statistics of outcome and predictor variables.

	Possible range	Actual range	Mean (SD)
Outcome variables			
Global HRQOL	0–4	2.00–3.96	3.03 (0.52)
Physical well-being	0–4	1.17–4.00	2.80 (0.82)
Functional well-being	0–4	0.71–3.86	2.55 (0.86)
Emotional well-being	0–4	2.33–4.00	3.39 (0.56)
Social well-being	0–4	1.60–4.00	3.40 (0.70)
Predictor variables			
Shoulder pain intensity	0–10	0–7.25	2.25 (2.34)
Shoulder pain distress	0–10	0–10.00	2.86 (3.36)
Shoulder abduction	0–180	60.00–158.00	111.59 (27.27)
Breathing impairment	0–4	0–4.00	0.76 (1.18)
Eating impairment	0–4	0–4.00	2.08 (1.11)
Speaking impairment	0–4	0–3.50	1.76 (1.01)
Impaired body image	0–4	0–4.00	1.38 (1.59)

HRQOL: health-related quality of life.

**Table 3:** Bivariate analyses among independent and outcome variables (Pearson *r*).

Independent variables	Outcome variables				
	Global HRQOL	Physical well-being	Functional well-being	Emotional well-being	Social well-being
Symptom experience					
Shoulder pain intensity	−0.20	−0.42^a^	−0.19	−0.17	0.26
Shoulder pain distress	−0.24	−0.49^a^	−0.21	−0.18	0.26
Functional status					
Shoulder abduction	0.27	0.35	0.46^a^	0.20	−0.38
Breathing impairment	−0.17	−0.06	0.05	−0.31	−0.24
Eating impairment	−0.42^a^	−0.45^a^	−0.53^b^	−0.20	−0.10
Speaking impairment	−0.32	−0.10	−0.56^b^	−0.15	−0.20
Impaired body image	−0.33	−0.23	−0.45^a^	−0.11	−0.07

HRQOL: health-related quality of life.

^a^ *P* < 0.05.

^b^ *P* < 0.01.

**Table 4:** Final models from the stepwise regression analyses

Outcome variables Independent variables	*R* ^2^	*B*	SE*b *	*t*	*P*
HRQOL^a^					
Symptom experience					
Physical well-being^b^	0.24				
(Constant)		3.15	0.18	17.55	<0.01
Shoulder pain distress		−0.12	0.41	−2.91	0.01
HRQOL^c^					
Functional status					
Global HRQOL^d^	0.18				
(Constant)		3.44	0.19	18.03	<0.01
Eating impairment		−0.20	0.08	−2.41	0.02
Physical well-being^e^	0.21				
(Constant)		3.50	0.30	11.79	<0.01
Eating impairment		−0.34	0.13	−2.65	0.01
Functional well-being^f^	0.45				
(Constant)		3.58	0.26	13.61	<0.01
Speaking impairment		−0.43	0.13	−3.41	<0.01
Impaired body image		−0.20	0.08	−2.55	0.02

HRQOL: health-related quality of life.

^a^No statistically significant associations were found for symptom experience variables with global HRQOL, functional well-being, emotional well-being, or social well-being outcome variables.

^b^Shoulder pain intensity was dropped from the model.

^c^No statistically significant associations were found for functional status variables with emotional well-being or social well-being outcome variables.

^d^Shoulder abduction, breathing impairment, speaking impairment, and impaired body image were dropped from the model.

^e^Shoulder abduction, breathing impairment, speaking impairment, and impaired body image were dropped from the model.

^f^Shoulder abduction, breathing impairment, and eating impairment were dropped from the model.
